# Suppressing NiO_x_/CsPbIBr_2_ Interfacial Redox Reactions and Band Energy Misalignment in Perovskite Solar Cells

**DOI:** 10.1002/smtd.202501684

**Published:** 2026-01-29

**Authors:** Xingnan Qi, Jiantao Wang, Baichuan Dong, Weihai Zhang, Heng Liu, Augusto Amaro, Bo Yang, Shuang Qiu, Makhsud I. Saidaminov, Hsing‐Lin Wang

**Affiliations:** ^1^ Materials Science and Engineering Southern University of Science and Technology Shenzhen Guangdong China; ^2^ Department of Electrical & Computer Engineering University of Victoria Victoria British Columbia Canada; ^3^ School of New Energy Ningbo University of Technology Ningbo China; ^4^ Henan Key Laboratory of Advanced Conductor Materials Institute of Materials Henan Academy of Sciences Zhengzhou China; ^5^ Department of Chemistry University of Victoria Victoria British Columbia Canada; ^6^ Institute of Fundamental and Frontier Science University of Electronic Science and Technology of China (UESTC) Chengdu China; ^7^ State Key Laboratory of Quantum Functional Materials Southern University of Science and Technology Shenzhen China

**Keywords:** band energy mismatch, crystallization, CsPbIBr_2_, perovskite solar cells, redox reactions

## Abstract

Inverted inorganic CsPbIBr_2_ perovskite solar cells (PSCs) employing NiO_x_ as the hole‐transport layer are promising long‐term stable and semi‐transparent photovoltaic devices. Nevertheless, their performance is often constrained by unfavorable interfacial phenomena, including detrimental redox reactions between Ni^3+^ in NiO_x_ and I^−^ in perovskite, as well as band energy misalignment at the NiO_x_/CsPbIBr_2_ interface. In this work, we introduce an N‐dodecylphosphonic acid (NDPA) interfacial modification strategy, where the phosphonic groups of NDPA anchor onto the NiO_x_ surface. This tailored interface not only suppresses interfacial redox reactions but also alleviates energy level mismatch and releases the residual tensile stress, thereby facilitating charge transport and reducing non‐radiative recombination losses. As a result, the optimized PSCs deliver a champion power conversion efficiency of 9.28% with a high open‐circuit voltage (*V*_oc_) of 1.12 V, positioning it among the best‐performing inverted CsPbIBr_2_ PSCs reported to date. The modified devices retain 79% of their initial efficiency after 1300 h of storage in a nitrogen‐filled glovebox, underscoring their potential for practical photovoltaic applications.

## Graphical abstract

High‐performance inverted inorganic CsPbIBr_2_ perovskite solar cells are achieved by introducing a N‐dodecylphosphonic acid interlayer at the NiO_x_/CsPbIBr_2_ interface.

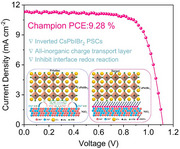

## 1 Introduction

Perovskite solar cells (PSCs) have attracted significant attention due to their tunable bandgap, high light absorption coefficient, low‐cost solution‐processable fabrication, and high power conversion efficiency (PCE) of over 27% [1, 2, 3, 4]. However, the instability of organic cations (e.g., methylammonium MA^+^, formamidinium FA^+^) poses a major challenge for their commercialization [5, 6]. All‐inorganic PSCs, particularly those based on cesium lead halide (CsPbX_3_, X = Cl, Br, I) compositions, have emerged as promising candidates owing to their superior thermal stability and intrinsic resistance to moisture compared to their organic–inorganic hybrid counterparts [7, 8, 9]. Among them, CsPbI_3_ possesses a suitable photovoltaic bandgap of ∼1.73 eV but suffers from crystal phase instability, as the photoactive α‐phase readily converts to the non‐photoactive δ‐phase at room temperature [10]. In contrast, CsPbBr_3_ exhibits excellent phase stability but achieves lower PCE due to its high bandgap (2.3 eV) [11, 12].

Mixed‐halide compositions (CsPbI_x_Br_3‐x_) have emerged as promising candidates to balance stability and efficiency [13, 14]. Especially, CsPbIBr_2_ perovskites are particularly attractive for semi‐transparent photovoltaics and tandem solar cells due to their wide bandgap (∼2.05 eV) and high optical transparency. Despite their potential, research on CsPbIBr_2_ for inverted‐structured (*p‐i‐n*) devices remains limited, hindering its application in all‐inorganic and tandem solar cells [15]. The development of inverted CsPbIBr_2_ PSCs has been further challenged by high crystallization temperatures, poor film quality, and significant interface barriers [16]. Inverted architectures employing NiO_x_ as a hole‐transport layer (HTL) have drawn significant attention because NiO_x_ offers high transparency, suitable valence band alignment, excellent chemical stability, and compatibility with low‐temperature processing [17, 18, 19]. Despite these advantages, the performance of inverted inorganic perovskite devices based on NiO_x_ is often hampered by severe challenges [20, 21, 22, 23]. The quality of NiO_x_ films significantly impact the performance of PSCs. Wang et al. removed impurities (such as nitrate ions) from NiO_x_ film employing an ionic liquid‐assisted method, thereby achieving higher conductivity and enhanced hole‐extraction ability, boosting PCE and stability of the modified‐devices [24]. These limitations stem mainly from uncoordinated metal ion defects and oxygen vacancies in NiO_x_ films. The higher Ni^3+^/Ni^2+^ ratio critically influences charge conductivity in NiO_x_ HTLs [25]. However, uncoordinated Ni^3+^ species can react detrimentally with perovskite components through dual roles as Bronsted proton acceptors and Lewis electron acceptors. As demonstrated by McGehee et al., this interaction leads to deprotonation of cationic amines and oxidation of iodide species, forming PbI_2‐x_Br_x_‐rich interfacial phases [26]. Wu et al. introduced redox‐sensitive NiO_x_ nanoparticles into antisolvent, thus repairing Pb^0^ and I^0^ defects by Ni^3+^/Ni^2+^ redox reactions, which yielded a 22.9% module PCE [27]. Our previous work further revealed redox reactions between Ni^3+^ and Sn^2+^ at the interface, which could be suppressed by 4‐ABSA incorporation, enabling 17.4% PCE in all‐inorganic Pb‐Sn PSCs [28]. Additionally, the ‐5.3 eV valence band maximum (VBM) of NiO_x_ creates a significant energy barrier when paired with CsPbIBr_2_ (VBM ∼ ‐5.9 eV) [29, 30]. severely impeding charge transfer and leading to substantial non‐radiative recombination. This mismatch results in reduced *V*_oc_ and overall device performance [31]. A Previous study by Ma et al. addressed this issue through N749 interfacial modification, achieving 9.49% PCE in NiO_x_‐based CsPbIBr_2_ PSCs. Notably, a weak binding energy of the carboxyl group of N749 may cause a desorption from the NiO_x_ surface [32]. Therefore, developing strategies to simultaneously suppress detrimental interfacial redox reactions and optimize energy level alignment would be crucial for fabricating highly efficient and stable NiO_x_‐based inverted CsPbIBr_2_ PSCs.

In this work, we introduce N‐dodecylphosphonic acid (NDPA), featuring both phosphonic (‐PO(OH)_2_) anchoring group and a carbon chain, as an effective modifier for NiO_x_ to optimize its performance as an HTL in inorganic CsPbIBr_2_ PSCs. The ‐PO(OH)_2_ group in NDPA strongly coordinates to NiO_x_, increasing the Ni^3+^/Ni^2+^ ratio and enhancing film conductivity. Simultaneously, the carbon chain acts as a physical barrier, preventing direct contact between Ni^3+^ species and I^−^ ions and thereby suppressing detrimental redox reactions. Furthermore, NDPA can effectively improve perovskite crystallinity, release residual tensile stress, and mitigate energy level misalignment at the NiO_x_/perovskite interface. These synergistic effects enable NiO_x_‐based inverted CsPbIBr_2_ PSCs to achieve a champion power conversion efficiency (PCE) of 9.28% with a high open‐circuit voltage (*V*_oc_) of 1.12 V. Notably, the NDPA‐modified devices retain 79% of their initial PCE after storing 1 300 h in a glovebox, demonstrating exceptional stability.

## 2 Results and Discussion

**FIGURE 1:**
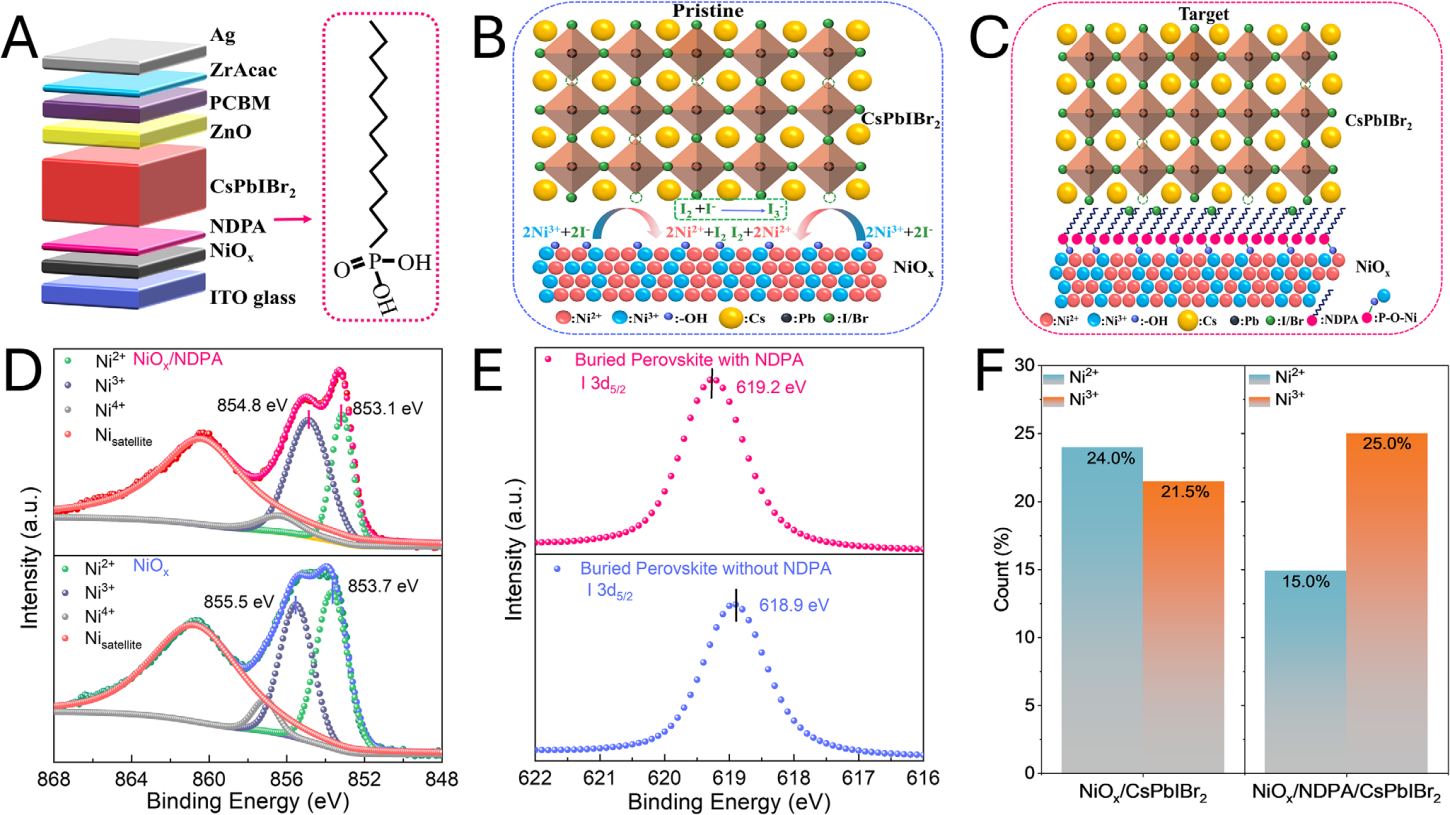
(A) schematic diagram of inverted CsPbIBr_2_ PSC; (B,C) schematic of interactions between NiO_x_ layer and CsPbIBr_2_ of (B) pristine sample (without NDPA) and (C) target sample (with NDPA); (D) XPS spectra of the Ni 2p on NiO_x_ and NiO_x_/NDPA, and corresponding changes with the deposition of CsPbIBr_2_; (E) XPS spectra of the I 3d on buried pristine and target CsPbIBr_2_ films after delamination from HTL/perovskite interface; (F) concentration of Ni^2+^, Ni^3+^ on NiO_x_ and NiO_x_/NDPA extracted from XPS spectra.

We first fabricated inverted inorganic CsPbIBr_2_ PSCs using NiO_x_ as HTL (Figure 1a), which we denote as the pristine sample hereafter. For the target sample, N‐dodecylphosphonic acid (NDPA) was spin‐coated onto NiO_x_. The chemical interaction mechanism is summarized in Figure 1b,c: an unfavorable redox reaction may occur between Ni^≥3+^ and I^−^ to form Ni^2+^ and I_2_ during the CsPbIBr_2_ film formation on NiO_x_ HTL. This redox reaction negatively impacts both the conductivity of NiO_x_ HTL and the crystallization of CsPbIBr_2_ perovskite films, causing lower device photovoltaic performance [28]. This redox reaction is significantly suppressed by the strongly coupled interaction between the ‐PO(OH)_2_ group in NDPA with NiO_x_ HTL and physical barrier (carbon chain), as schematized in Figure 1c [17]. With these apparent advantages, it is expected that the NDPA functional layer can improve the efficiency and stability of target CsPbIBr_2_ PSCs.

To track the occurrence of described redox reactions, we first deposited a thin layer of CsI (as I^−^ source) on NiO_x_ and performed X‐ray photoelectron spectroscopy (XPS) (Figures S1–S3). The Ni 2p_3/2_ core‐level spectra revealed four characteristic peaks at 853.18 eV (Ni^2+^), 854.98 eV (Ni^3+^), 856.28 eV (Ni^4+^), and 860.18 eV (satellite peak) [26]. Quantitative analysis showed the relative proportions of these oxidation states on pristine NiO_x_ to be 25.4% (Ni^2+^), 26.6% (Ni^3+^), and 5.7% (Ni^4+^) (Figure S2). Upon CsI deposition, we observed a significant redox response: [Ni^2+^] increased to 31.7%, while [Ni^3+^] decreased to 18.5% due to the following reactions:

(1) $$2Ni^{3+}+2I^{-}=2Ni^{2+}+I_{2}$$

(2) $$I_{2}+I^{-}=I_{3}^{-}$$

This reduction in [Ni^3+^] detrimentally affects NiO_x_ conductivity, thereby impairing hole transport at the HTL/perovskite interface [28, 33]. To mitigate this, we introduced NDPA (structure shown in Figure 1a) onto the NiO_x_ surface through phosphonic group anchoring. XPS analysis (Figures S1–S3) revealed that after CsI deposition, the [Ni^3+^] decreased by only 2.6% (from 27.5% to 24.9%) on target ITO/NiO_x_/NDPA, which is three‐fold smaller than 8.1% (from 26.6% to 18.5%) on pristine ITO/NiOx. This suppression in the interface redox reaction will help in maintaining optimal charge transport properties at NiO_x_/perovskite interface, as seen below.

We then also tracked the occurrence of these redox reactions at the NiO_x_/perovskite interface after delaminating perovskite by a UV‐curing adhesive (Figure S4). The presence of P (133 eV) 2p_3/2_ XPS peak on target NiO_x_/NDPA (Figure S5 and S6) confirmed that NDPA binds to the surface of NiO_x_. The Ni^2+^ (853.7 eV) and Ni^3+^ (855.5 eV) 2p_3/2_ XPS peaks for pristine NiO_x_ shifted to 853.1 eV (Ni^2+^) and 854.8 eV (Ni^3+^) for target NiO_x_/NDPA, respectively, (Figure 1d and Figure S6), indicating change in Ni environment due to chemical bonding between Ni and ‐PO(OH)_2_ of NDPA [26]. [Ni^3+^] was higher (25.0% vs 21.5%) while [Ni^2+^] was lower (24.0% vs 15.0%) on target NiO_x_/NDPA vs pristine NiO_x_ (Figure 1f), indicating suppressed reduction of Ni^3+^ in the presence of NDPA. Additionally, I (618.9 eV) 3d_5/2_ XPS peak detected on the surface of buried pristine CsPbIBr_2_ film shifted from to 619.2 eV on the target film (Figure 1e) [34], indicating a change in iodine chemical environment due to the suppression of the oxidation of I^−^.

The anchoring behavior of the ‐PO(OH)_2_ group in NDPA on NiO_x_ was studied by Fourier transform infrared spectroscopy (FTIR) (Figure S7). The characteristic peaks of P═O (1074 cm^−1^) and P‐OH (1002, 952 cm^−1^) from NDPA shifted when deposited on NiO_x_ to 1046 cm^−1^ for P═O and 999, 938 cm^−1^ for P‐OH [35], indicating NDPA anchoring to NiO_x_.

The occurrence of the redox reactions was further examined by UV–vis absorption spectroscopy of toluene solution in which NiO_x_/CsI and NiO_x_/NDPA/CsI samples were immersed for 24 h (Figure S8). As expected, characteristic I_2_ and I_3_^−^ absorption peaks [36, 37] were observed for the NiO_x_/CsI sample, indicating that Ni^3+^ and I^−^ in NiO_x_/CsI interact to generate iodine (Equation 2) [37]. In contrast, the NiO_x_/NDPA/CsI sample exhibited negligible iodine‐related adsorption, implying that NDPA acts as a physical barrier to suppress direct interaction between Ni^3+^ and I^−^.

The adverse impact of the redox reactions on charge transport at the NiO_x_/CsI interface was then studied via electrical conductivity measurement (Figures S9 and S10 and Table S1). The target NiO_x_/NDPA sample showed the highest conductivity (5.58 × 10^−6^ S cm^−1^) compared to pristine NiO_x_ (2.32 × 10^−6^ S cm^−1^), NiO_x_/CsI (2.22 × 10^−6^ S cm^−1^), and NiO_x_/NDPA/CsI substrate (3.87 × 10^−6^ S cm^−1^) [28], which can be attributed to an increase in hole mobility of NiO_x_ from 2.09 × 10^−5^ to 2.63 × 10^−5^ cm^2^ V^−1^ s^−1^ upon incorporation of NDPA (Figure S11) [38].

**FIGURE 2:**
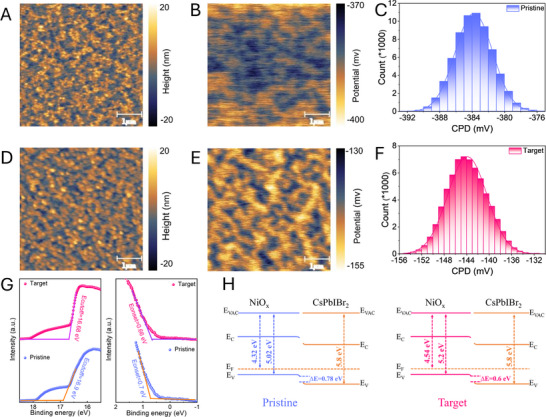
(A–F) the surface morphology, potential distribution, and corresponding histogram distribution images on NiO_x_ and on NiO_x_/NDPA substrate, respectively; (G) UPS spectra of NiO_x_ and on NiO_x_/NDPA substrate; H, the energy level diagram of NiO_x_‐based HTL with CsPbIBr_2_ film.

The surface morphology of the films were examined via scanning electron microscopy (SEM) (Figure S12). The NiO_x_/NDPA‐modified film showed a uniform and compact coverage on the substrate than the pristine film, which decreases the leakage current and suppresses recombination probabilities of charge carriers at the NiO_x_/CsPbIBr_2_ interface via promoting the vertical growth of CsPbIBr_2_ film [39]. The changes in morphology were quantified by atomic force microscope (AFM): the derived root mean square (RMS) roughness were 5.1 and 4.2 nm for pristine and target samples, respectively (Figure 2a,d). The reduced RMS is attributed to the anchoring effect of –PO(OH)_2_ group in NDPA on the NiO_x_ surface.

Subsequently, the Kelvin probe force microscopy (KPFM) was performed to assess the photoelectronic properties of the films. The pristine film (Figure 2b) presented a nonuniform surface potential distribution compared to the target film (Figure 2e), indicating that the surface chemical state of Ni was changed via phosphonic group anchoring [40, 41]. The derived average contact potential distribution (CPD) values of pristine and target films were ‐384 and ‐144 mV, respectively, indicating NDPA can decrease the work function (WF) (Figure 2c,f) [42].

To quantitatively evaluate the influence of the NDPA modification layer on the energy level and WF, ultraviolet photoelectron spectroscopy (UPS) was conducted. The corresponding valence band maximum (VBM) position (Figure 2g), the conduction band minimum (CBM) position, and the optical bandgap were confirmed from UPS and the Tauc plot spectra (Figure S13). With NDPA, the VBM of target NiO_x_ was at ‐5.2 eV, lower than that of pristine film (‐5.02 eV). The corresponding CBM positions were at ‐1.86 and ‐1.78 eV, respectively. The slight shift in the optical bandgap of the target film (3.34 eV) was due to modification of the surface chemical states via the anchoring effect of the phosphonic groups compared to the pristine film (3.24 eV).

The band energy levels between the NiO_x_ and CsPbIBr_2_ perovskite film is plotted in Figure 2h. The band energy levels of CsPbIBr_2_ film measured in our previous work were used here [43]. The energy level barrier between NiO_x_ and CsPbIBr_2_ film was reduced from 0.78 to 0.6 eV. Additionally, the WF was also calculated to be ‐4.32 (pristine film) and ‐4.54 eV (target film) from UPS, which agrees with the KPFM discussed above. The increased NiO_x_ film quality and decreased energy mismatch can facilitate charge carrier transfer and suppress the carrier non‐radiative recombination at NiO_x_/CsPbIBr_2_ interface [32].

**FIGURE 3:**
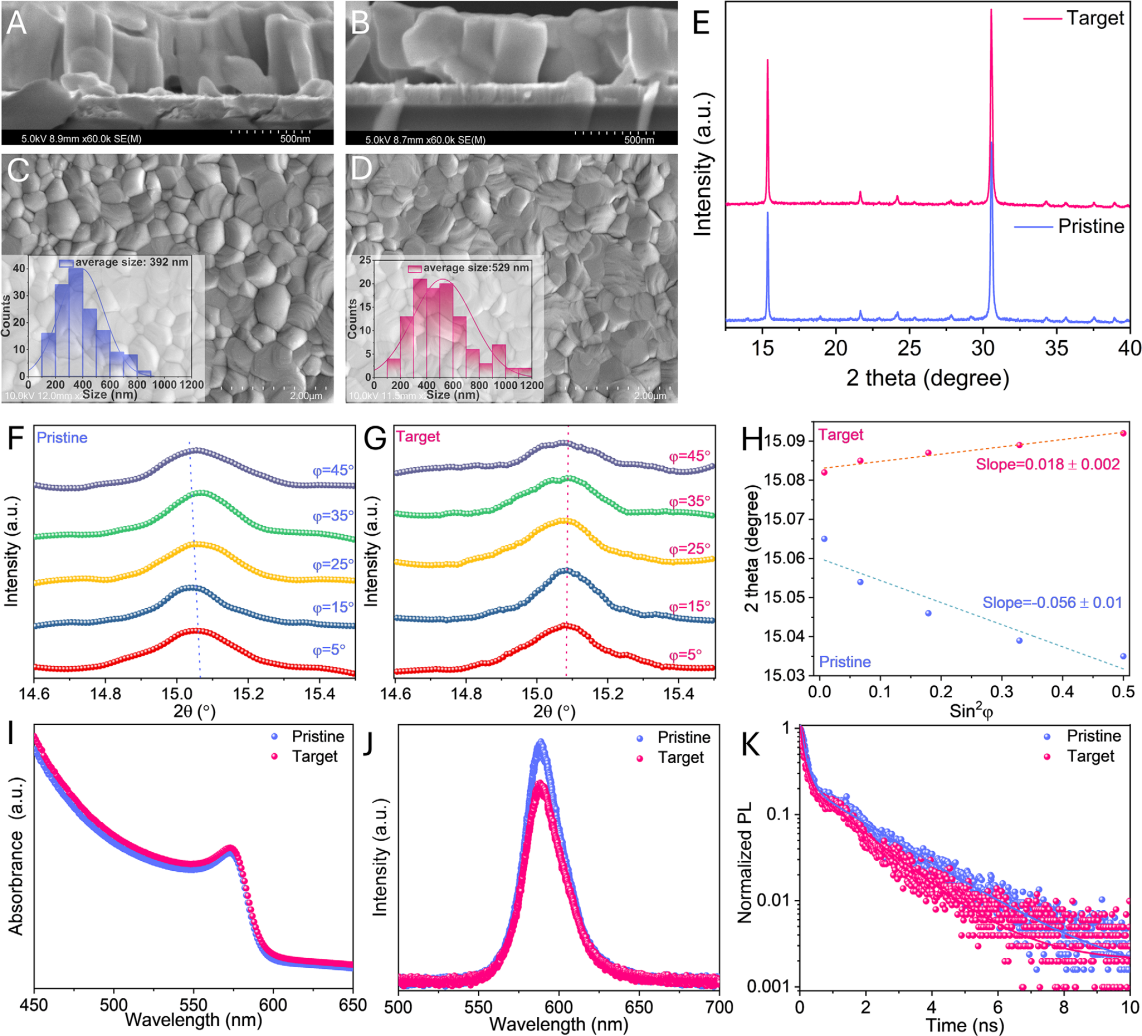
(A–D) cross‐section and top surface SEM images of CsPbIBr_2_ films on pristine and target substrates. Insets in panels (C,D) exhibit the grain size distribution; (E) XRD profiles of CsPbIBr_2_ films on pristine and target substrates; (F,G) GIXRD spectra of perovskite films on pristine and target substrates and (H) corresponding fitting slope of GIXRD data; (I) UV–vis spectra; (J) PL spectra and (K) TRPL decay of the CsPbIBr_2_ films on pristine and target substrates.

The structural and optoelectronic properties of CsPbIBr_2_ films on pristine and target substrates were then investigated (the fabrication steps are detailed in the experimental section). The film morphologies were observed from both the top surface and cross‐section SEM. In contrast to small and irregular grains in pristine sample, the target film showed large and vertically‐oriented CsPbIBr_2_ grains (Figure 3a,b), which would facilitate charge carrier transport as needed for operational devices [44]. Figure 3c displays a uniform surface morphology with some extra small grains on pristine substrate, whereas the CsPbIBr_2_ film grown on target substrate shows an enlarged grain size (Figure 3d). The grains in CsPbIBr_2_ films increased from 392 (pristine substrate) to 529 nm (target substrate) as seen in the insets of Figure 3c,d: this reveals that NDPA modification imposes a positive impact on the crystallization of CsPbIBr_2_ films.

X‐ray diffraction (XRD) on pristine CsPbIBr_2_ exhibited two main diffraction peaks at 15.41° and 30.66°(Figure 3e), corresponding to the (100) and (200) crystallographic planes of α‐phase in CsPbIBr_2_ perovskite, respectively [45]. The peak intensity of the peaks became stronger on the target substrate, as expected from increased grains.

Grazing‐incidence X‐ray diffraction (GIXRD) was utilized to study the NDPA influence on the film's residual stress using Bragg's Law and generalized Hook's Law [2]:

(3) $$\sigma =-\frac{E_{p}}{\left(1+v_{p}\right)}\frac{\pi }{180^{\circ }}Cot\theta_{0}\frac{\partial \left(2\theta \right)}{\partial \left[{\left(sin\varphi \right)}^{2}\right]}$$

where *E_p_* is the perovskite modulus (10 GPa), *v_p_* is Poisson's ratio of the perovskite (0.3) [46], θ_0_ is half of the scattering angle 2θ for stress‐free perovskite (2θ_0_ = 15.4°). For GIXRD measurement, the (100) crystal plane located at 15.4° was selected. In contrast to target film, the diffraction peaks of the pristine film gradually shifted to a lower 2θ position by changing *φ* from 5° to 45° (Figure 3f,g). Figure 3h illustrates the fitting slopes of the diffraction peaks of pristine (‐0.056) and target film (0.018), so that the residual stress changed from 56.1 MPa (tensile stress) to ‐17.9 MPa (compressive stress). These results indicate that NDPA can observably release the residual tensile stress of CsPbIBr_2_ perovskite film, causing a higher efficiency and stability of target devices [47].

Optical properties of perovskite films were further studied by UV–vis, steady state photoluminescence (PL), and time‐resolved photoluminescence (TRPL) spectroscopy. The slightly increased absorption of CsPbIBr_2_ films on the target substrate indicates better light absorption ability due to enhanced crystallization (Figure 3i) [34, 48]. The PL peak intensity on the target substrate was lower than that on the pristine substrate, which means the NDPA modification can improve charge carrier extraction at the NiO_x_/CsPbIBr_2_ interface (Figure 3j) [49]. The CsPbIBr_2_ film on the target substrate also showed a shorter TRPL decay (1.1 ns) than on the pristine substrate (1.8 ns) (Figure 3k; Table S2), exhibiting a more effective charge extraction at the NiO_x_/CsPbIBr_2_ interface with the assistance of NDPA [33].

To investigate the influence of NDPA modification on photovoltaic performance, inverted PCSs were fabricated (Figure 1a). First, the bi‐layer electron transport layer (ETL) was optimized to obtain efficient CsPbIBr_2_ PSCs. Zinc oxide (ZnO) and phenyl‐C61‐butyric acid methyl ester (PCBM) were selected due to their high electron mobility and matched energy level at CsPbIBr_2_/ETL interface [50, 51]. The detailed *J*–*V* curves and photovoltaic parameters are displayed in Figure S14 and Table S3.

**FIGURE 4:**
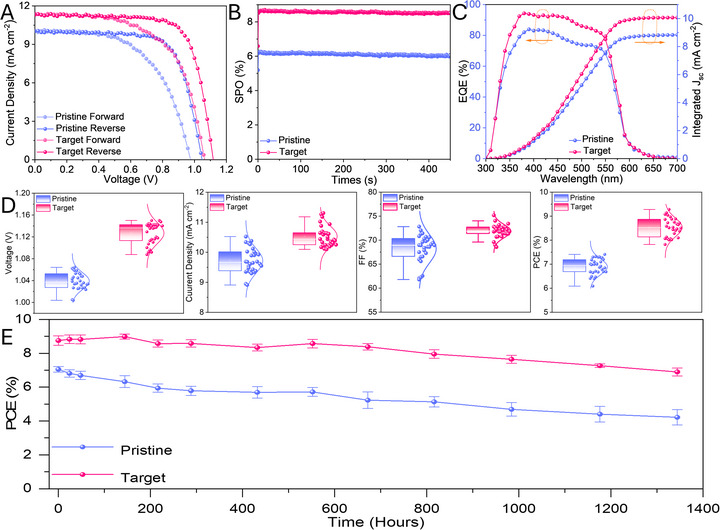
(A) *J–V* curves of the champion pristine and target PSCs; (B) SPO of pristine and target PSCs; (C), EQE spectra and integrated current of pristine and target PSCs; (D), performance of three individual pristine and target PSCs; (E) the PCE of pristine and target PSCs as stored in an inert condition.

Figure 4a displays characteristic *J–V* curves of champion PSCs for pristine and target samples. The detailed photovoltaic parameters are summarized in Table 1. The pristine device exhibited a PCE of 7.41%, with *V*_oc_ of 1.05 V, *J_sc_* of 10.0 mA cm^−2^, and FF of 70.6%, whereas the PCE of the target device has reached 9.28%, with *V*_oc_ of 1.12 V, *J*_sc_ of 11.3 mA cm^−2^, and FF of 73.4%. We brought together reported photovoltaic performance of NiO_x_‐based devices in Table S4, demonstrating that our results rank among the best‐performing inverted CsPbIBr_2_ PSCs reported to date. Hysteresis index (HI) was calculated following this relation [34]:

(4) $$\mathrm{HI}\;=\frac{\mathrm{PC}E_{\mathrm{reverse}}-\mathrm{PC}E_{\mathrm{forward}}}{\mathrm{PC}E_{\mathrm{reverse}}}$$

**TABLE 1:** The detailed photovoltaic parameters of the pristine and target devices.

Sample	*V*_oc_ (V)	*J*_sc_ (mA cm^−2^)	FF (%)	PCE (%)	HI (%)
Pristine (RS)	1.05	10.0	70.7	7.41	
Pristine (FS)	1.00	10.0	56.6	5.56	25.0
Target (RS)	1.12	11.3	73.4	9.28	
Target (FS)	1.06	11.4	60.8	7.37	20.6

*Note*: RS is reverse scan, FS is forward scan.

The target PSC possessed a lower HI (20.6%) than that of the pristine device (25.0%). The higher PCE and lower HI for target devices can be attributed to improved charge extraction at the NiO_x_/CsPbIBr_2_ interface and suppressed non‐radiative recombination within devices [33].

The steady‐state maximum power output (SPO) was performed and displayed in Figure 4b: the increased SPO from 8.65% (target) to 6.25% (control) confirms that NDPA can promote the operational stability of the target device. Moreover, the external quantum efficiency (EQE) and corresponding integrated current density were obtained from the champion devices. Figure 4c shows that the target device possessed an enhanced EQE spectral response compared to the pristine device. The corresponding integrated current densities are 10.1 and 8.83 mA cm^−2^, in relatively good agreement with *J–V* curve.

To assess the reproducibility of our devices, 30 individual devices were fabricated and characterized (Figure 4d). The average PCE increased from 6.89% (pristine devices) to 8.53% (target devices), with the average *V*_oc_, *J*_sc_, and FF reaching 1.13 V, 10.5 mA cm^−2^, and 71.9% from 1.04 V, 9.72 mA cm^−2^ and 68.2%, respectively. All photovoltaic parameters of target devices not only demonstrate an increasing trend but also have a narrow distribution compared to pristine devices.

The stability of pristine and target devices was assessed (Figure 4e). The target device retained 79% of its initial PCE after 1300 h of storage in an inert atmosphere; however, the counterpart decreased to 59% of its initial performance over the same period.

**FIGURE 5:**
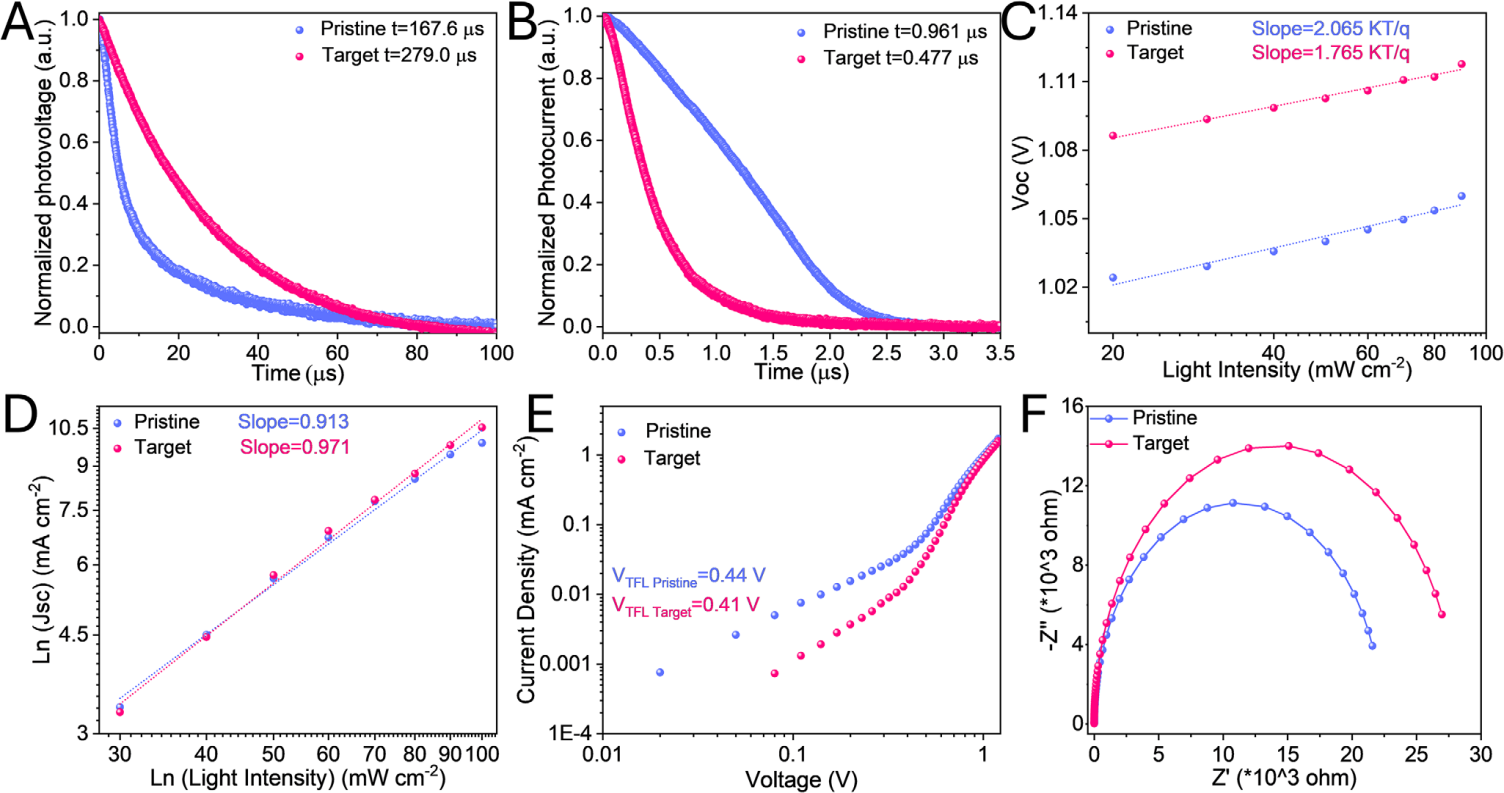
(A) TPV and (B) TPC decays of pristine and target PSCs; (C) *V*_oc_ and (D), *J_sc_* to the light intensity plots of pristine and target PSCs; (E) SCLC of electron‐only pristine and target half‐cells (ITO/SnO_2_/CsPbIBr_2_/ZnO/PCBM/Ag); (F) Nyquist plots for the pristine and target PSCs.

To assess the charge transport dynamics in PSCs, we performed various optoelectronic measurements. Figure 5a shows the transient photovoltage (TPV) results. The target PSC presented a longer charge recombination decay time of 279 µs than that of the pristine device (168 µs), demonstrating that the charge non‐radiative recombination is significantly suppressed within the target device [52]. Furthermore, the transient photocurrent (TPC) was utilized to assess charge extraction efficiency in pristine and target devices (Figure 5b). The target PSC showed a faster TPC decay (0.477 µs) than pristine PSC (0.961 µs), indicating an enhanced charge extraction efficiency at NiO_x_/CsPbIBr_2_ interface [53].

The *V*_oc_ as a function of light intensity (*P_light_*) was measured to study the charge recombination behavior for PSCs (Figure 5c). The relationship between *V*_oc_ and P_light_ can be described by the following equation [54]:

(5) $$n=\frac{q}{kT}\frac{dV_{oc}}{dlnP_{light}}$$

where *q, k, T*, and *n* represent electron charge, Boltzmann's constant, absolute temperature, and ideality factor, respectively. A lower *n* value (1.76) is attributed to lower trap‐assisted recombination within target PSCs compared to the pristine device (2.07). Meanwhile, the relationship between the current density and P_light_ (*J*_sc_ ∝ *P_light_^S^*) was also recorded and calculated (Figure 5d). The target device exhibits a more ideal S of 0.971 than the pristine device (0.913), which illustrates a higher charge extraction efficiency due to the optimal energy level alignment at NiO_x_/CsPbIBr_2_ interface as discussed with UPS results [55].

The trap‐state density (N_t_) for pristine and target devices was calculated according to the following equation (Figure 5e) [21]:

(6) $$N_{t}=\frac{2V_{TFL}\varepsilon_{r}\varepsilon_{0}}{qL^{2}}$$

where *L, ε_r_, ε_0_*, and *V_TFL_* are the thickness of CsPbIBr_2_ film, the relative dielectric (the approximate value is 8 for CsPbIbr_2_ film [43]), the vacuum permittivity, and the trap‐filled limit voltage of the devices. The V_TFL_ was reduced from 0.44 V (pristine device) to 0.41V (target device). Then the corresponding N_t_ decreased from 1.72 × 10^15^ to 1.59 × 10^15^ cm^−3^, which also confirms that NDPA effectively suppressed the carrier non‐radiative recombination in devices.

The typical Nyquist plot of the electrochemical impedance spectroscopy (EIS) spectra of pristine and target device at a 1V bias voltage under dark conditions were measured in Figure 5f (the fitting circuit is exhibited in Figure S15. The detailed fitting parameters of the insert equivalent circuit were displayed in Table S5, the charge transfer recombination resistance of the target device (2.8 × 10^4^ Ω) is much higher than that of the pristine device (2.2 × 10^4^ Ω), implying NDPA suppresses the carrier recombination and promotes photovoltaic performance [9].

## 3 Conclusion

In summary, we demonstrated NDPA as a multifunctional interfacial modifier that is able to enhance the photovoltaic performance of inverted inorganic NiO_x_‐based CsPbIBr_2_ PSCs. The results suggest that NDPA simultaneously suppresses detrimental interfacial redox reactions, optimizes energy level alignment, releases the residual stress of the bottom perovskite, and passivates buried interface defects. These combined effects synergistically enhance charge extraction efficiency while substantially suppressing non‐radiative recombination at the NiO_x_/CsPbIBr_2_ interface. As a result, the NDPA‐modified devices achieve a champion PCE of 9.28%, among the highest reported for inverted CsPbIBr_2_ PSCs, while demonstrating excellent long‐term stability. This work presents an effective strategy for fabricating highly efficient and stable NiO_x_‐based inorganic perovskite solar cells.

## Conflicts of Interest

The authors declare no conflict of interest.

## Supporting information




**Supporting File**: smtd70514‐sup‐0001‐SuppMat.pdf.

**Supporting File**: smtd70514‐sup‐0002‐DataFile.zip.

## Data Availability

The data that support the findings of this study are available in the supplementary material of this article.
