# Supplementary material for: Suppressing NiOx/CsPbIBr2 Interfacial Redox Reactions and Band Energy Misalignment in Perovskite Solar Cells
Source: Small Methods. 2026 Jan 29;10(4):e01684. doi: 10.1002/smtd.202501684 (PMC12929923; doi:10.1002/smtd.202501684)
Supplement: Supplementary file 1 — Supporting File: smtd70514‐sup‐0001‐SuppMat.pdf. [file SMTD-10-e01684-s002.pdf]

Supporting Information

Suppressing $NiO_x/CsPbIBr_2$ Interfacial Redox Reactions and Band Energy Misalignment in Perovskite Solar Cells

Xingnan Qi[1,2+], Jiantao Wang[1+], Baichuan Dong[1], Weihai Zhang[3]*, Heng Liu[4], Augusto Amaro[5], Bo Yang[6], Shuang Qiu[5], Makhsud I. Saidaminov[2,5]*, Hsing-Lin Wang[1,7]*

[1]Materials Science and Engineering, Southern University of Science and Technology, Shenzhen 518055, Guangdong Province, China

[2]Department of Electrical & Computer Engineering, University of Victoria, 3800 Finnerty Road, Victoria, BC V8P 5C2, Canada

[3]School of New Energy, Ningbo University of Technology, Ningbo, 315336, China

[4]Henan Key Laboratory of Advanced Conductor Materials, Institute of Materials, Henan Academy of Sciences, Zhengzhou 450046, China

[5]Department of Chemistry, University of Victoria, Victoria, British Columbia V8P 5C2, Canada

[6]Institute of Fundamental and Frontier Science, University of Electronic Science and Technology of China (UESTC), Chengdu 610054, China

[7]State Key Laboratory of Quantum Functional Materials, Southern University of Science and Technology, Shenzhen 518055, China.

[+]These authors contributed equally to this work.

*E-mails:

zhangwh@nbut.edu.cn

msaidaminov@uvic.ca (MIS)

wangxl3@sustech.edu.cn

Contents

Experimental section: 3

Figure S1. XPS spectra of the Ni 2p on NiO_x and NiO_x/CsI substrates (left); on NiO_x/NDPA and NiO_x/NDPA/CsI substrates (right). 5

Figure S2. (a) Concentration of Ni^{2+}, Ni^{3+} and Ni^{4+} on NiO_x and NiO_x/CsI substrates; (b) on NiO_x/NDPA and NiO_x/NDPA/CsI substrates extracted from XPS spectra. 6

Figure S3. (a,b) Survey XPS spectra of the different NiO_x-based films. 7

Figure S4. The mechanism of delamination method to measure Ni 2p, I 3d and P 2p XPS spectra on pristine and target substrates. 8

Figure S5. XPS spectra of P 2p on NiOx and NiO_x/NDPA substrates. 9

Figure S6. Survey XPS spectra on NiO_x and NiO_x/NDPA substrates. 10

Figure S7. FTIR spectra of the pure NDPA solution and the mixing between NDPA and NiO_x. 11

Figure S8. Solution UV-Vis spectra of the I_2, NiO_x/CsI and NiO_x/NDPA/CsI substrates in toluene after storing for 24 hours. 12

Figure S9. Current-voltage characteristics of NiO_x-based substates. 13

Figure S10. Thickness of NiO_x on different substrates. 14

Figure S11. The *J*-V characteristic of ITO/NiO_x without or with NDPA/Ag hole-extracting devices. 15

Figure S12. Surface morphology of NiO_x-based substates via SEM measurement... 16

Figure S13. UV-Vis spectra a) and the corresponding Tauc plots b) of the different NiO_x-based substates. 17

Figure S14. *J-V* curves of the $CsPbIBr_2$ PSCs using different ETL. 18

Figure S15. Fitting circuit of the EIS plots. 19

Table S1 Conductivity of NiO_x-based substrates. 20

Table S2 detailed fitting parameters of the pristine and target perovskite films. 20

Table S3 detailed photovoltaic parameters of different ETL-based $CsPbIBr_2$ PSCs.. 20

Table S4 the statistic photovoltaic performance for NiO_x-based $CsPbIBr_2$ perovskite solar cells. 20

Table S5 fitting parameters of the EIS plots. 21

Experimental section:

Materials: The ITO substrates, nickel oxide (NiO_x), [6,6]-Phenyl C61 butyric acid methyl ester (PCBM) were purchased from Advanced Election Technology Co., Ltd. Cesium iodide (CsI), was purchased from Xi'an Yuri Solar Co., Ltd. Lead bromide ($PbBr_2$) was purchased from TCI(Shanghai) Development Co. Ltd. Dimethyl sulfoxide (DMSO, 99.9%) was purchased from Sigma-Aldrich. All chemicals are utilized without further purification before using.

Device Fabrication: ITO glass substrates (7 Ω sq^{-1}) were sequentially cleaned by ethanol, acetone and isopropyl alcohol using ultrasonic and treated with plasma for 5 min before usage. Then, the NiO_x layer (10 mg/mL in DI water) was spin-coated at 4000 rpm for 30 s and annealed at 120 °C for 10 min in ambient air. Then the substrates were quickly transferred into the glove box for the subsequent usage. The NDPA (0.005 mg/mL in ethanol) interfacial layer was spin-coated at 4000 rpm for 30 s and annealed at 100 °C for 10 min. The $CsPbIBr_2$ inorganic perovskite films were spin-coated from 1.2 M (CsI: $PbBr_2$=1:1) precursor solution in DMSO, the spin protocol is 4000 rpm for 50 s using free anti-solution method. The as-prepared perovskite film was annealed using subsequent two-step annealing method at 60 °C for 1 min and 200 °C for 10 mins. After cooling to room temperature, the bi-electron transport layer for ZnO and PCBM were spin-coated on the surface of as-prepared perovskite films. The ZnO was synthesized using previous literature method,[1] then as-synthesized ZnO solution was spin-coated at 4000 rpm for 30 s, followed by annealing at 180 °C for 10 min. Then, 10 mg mL^{-1} PCBM in chlorobenzene was spin-coated at 1000 rpm for 40 s, followed by annealing at 70 °C for 5 min. The ZnO and PCBM bi-layer structure was used as the electron transport layer. After that, ZrAcac (1.5 mg mL^{-1} in ethanol) was further spin-coated by spin-coating at 5000 rpm for 40 s as the cathode interface layer without annealing process. Finally, 100 nm Ag was deposited on the top surface of the as-prepared substrate as the electrode using thermal evaporation system under higher vacuum. The active area of the devices was 0.06 cm^2.

Characterizations: X-ray photoelectron spectroscopy (XPS) was carried out on a Thermo Scientific^{TM} K-Alpha^{TM+} spectrometer equipped with a monochromatic Al Kα X-ray source (1486.6 eV) operating at 100 W. All characterized peaks were calibrated with C 1s peak binding energy at 284.8 eV for adventitious carbon. The XRD spectrum were measured on Bruker Advanced D8 X-ray diffractometer using Cu Kα (λ = 0.154 nm) radiation. The GIXRD spectrum were carried out using PANalytical Empyrean X-ray System. Ultraviolet photoelectron spectroscopy (UPS, ESCALAB 250Xi, Thermo Fisher) measurements were carried out using a He I discharge lamp (21.22 eV). A UV-Vis spectrophotometer (Agilent Cary 5000) was utilized to record the solution absorption spectrum of the NiO_x/CsI films in toluene and absorption spectrum of the perovskite films. Steady state photoluminescence (PL) spectra were recorded on Shimadzu RF-5301pc. Time-resolved photoluminescence (TRPL) spectra were conducted on a PL system (Fluo-Time 300) under excitation with a picosecond pulsed diode laser at 640 nm wavelength with a repetition frequency of 1 MHz. The morphology of the perovskite films and NiO_x-based films was carried out by field-emission scanning electron microscopy (FESEM, TESCAN, MIRA3) and atomic force microscopy (AFM, the MFP-3D SPM by Asylum Research). In addition, the surface potential distribution of the NiO_x film was also recorded by using this AFM system. Current density-voltage (J-V) curves of the devices were measured by using a source meter (Keysight B2901A) and a solar simulator (Enlitech SS-F5-3A) with a protocol of 1.2 to -0.2 V with a 20 mV voltage step and 10 ms delay. The external quantum efficiency (EQE) spectra were tested with a quantum efficiency measurement system (Enlitech QER-3011) in which the light intensity at every wavelength was calibrated with a Si detector before measurement. The stable power output (SPO) was recorded by our J-V curves measurement system. The electrochemical impedance spectroscopy analyses (EIS) were carried out with the frequency range from 100 Hz to 1M Hz on an electrochemical workstation (IM6ex, Zahner, Germany) in a bias voltage at 1 V under dark condition.

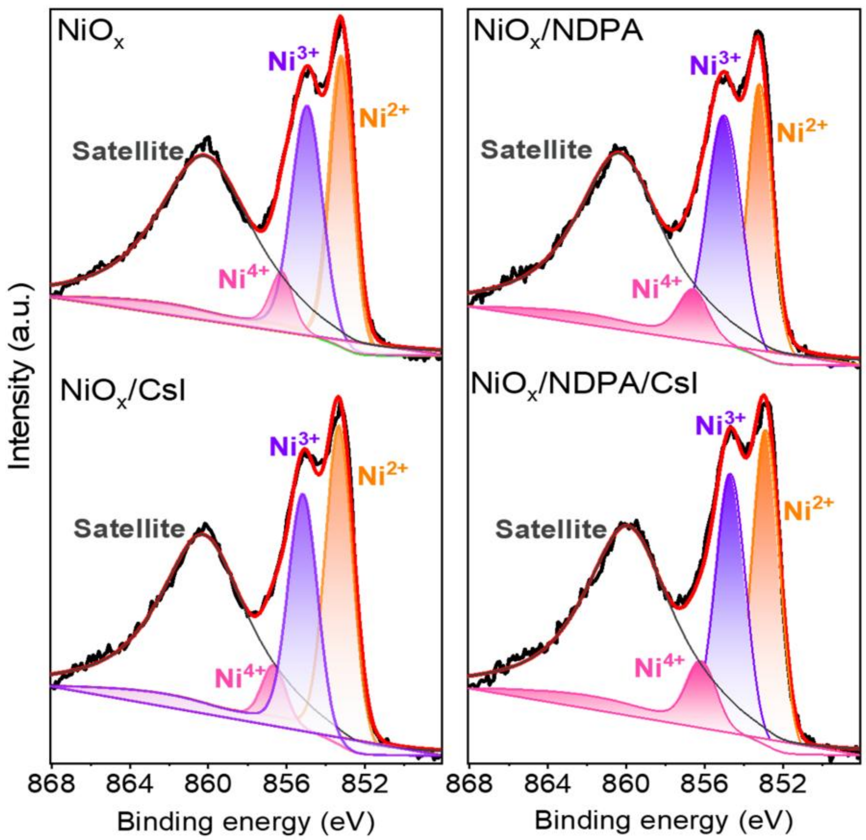

Figure S1. XPS spectra of the Ni 2p on NiO_x and NiO_x/CsI substrates (left); on NiO_x/NDPA and NiO_x/NDPA/CsI substrates (right).

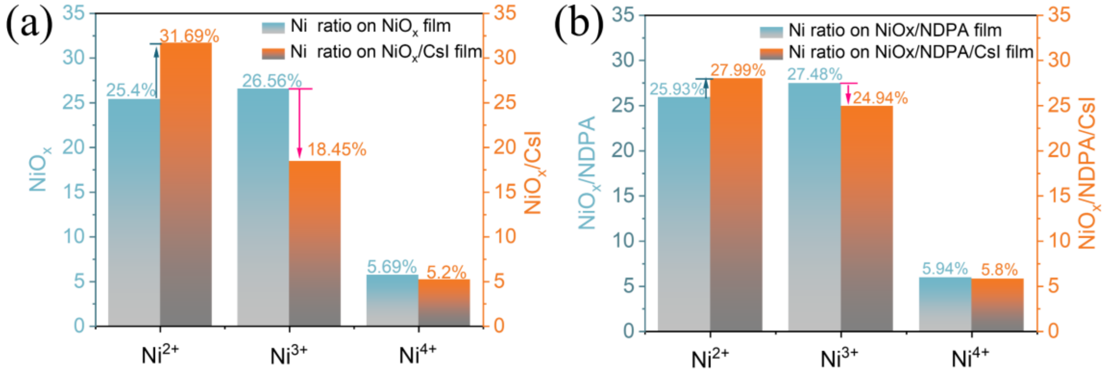

Figure S2. (a) Concentration of Ni^{2+}, Ni^{3+} and Ni^{4+} on NiO_x and NiO_x/CsI substrates; (b) on NiO_x/NDPA and NiO_x/NDPA/CsI substrates extracted from XPS spectra.

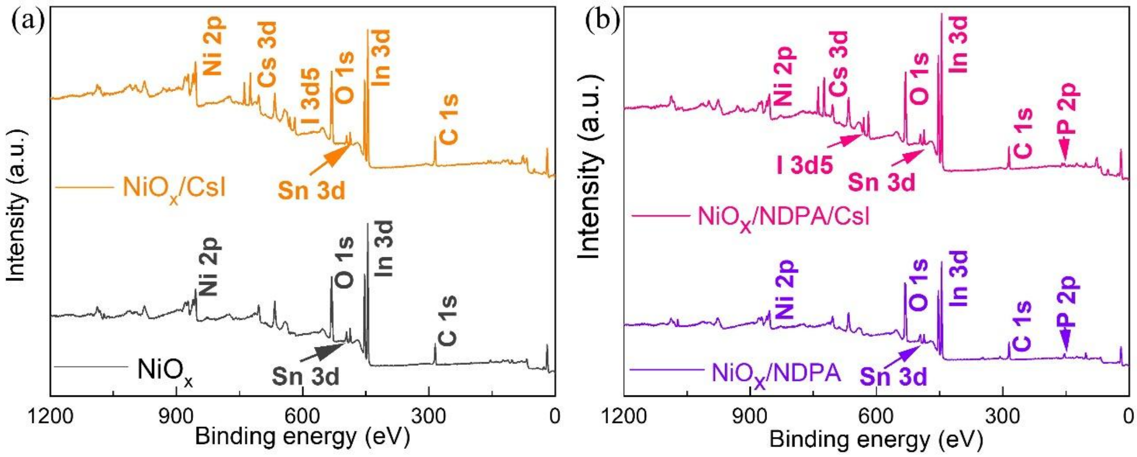

Figure S3. (a,b) Survey XPS spectra of the different NiO_x-based films.

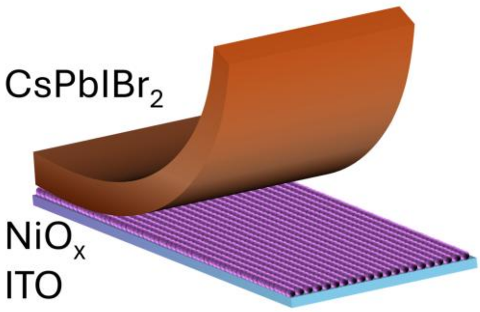

Figure S4. The mechanism of delamination method to measure Ni 2p, I 3d and P 2p XPS spectra on pristine and target substrates.

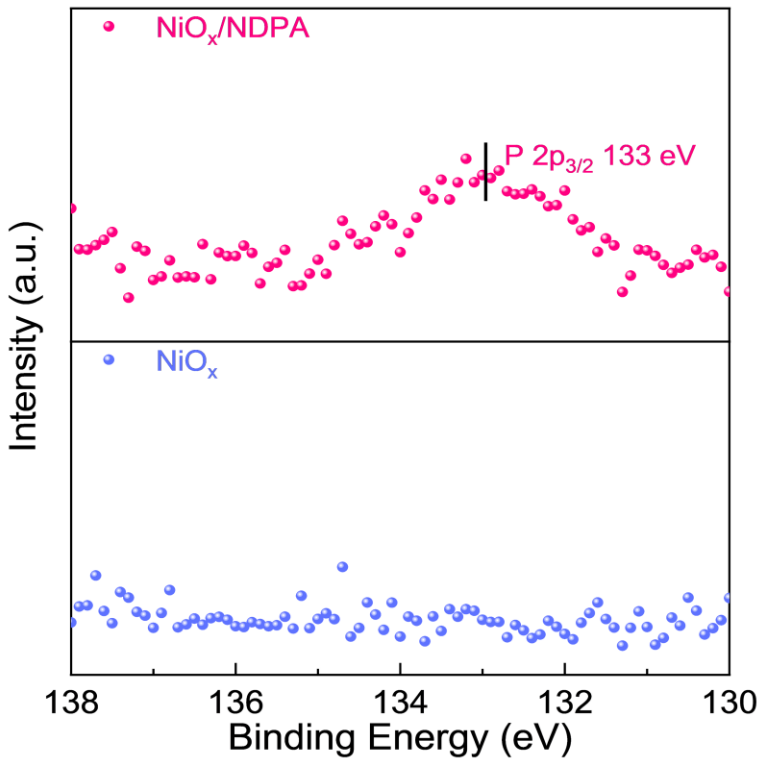

Figure S5. XPS spectra of P 2p on NiOx and NiO_x/NDPA substrates.

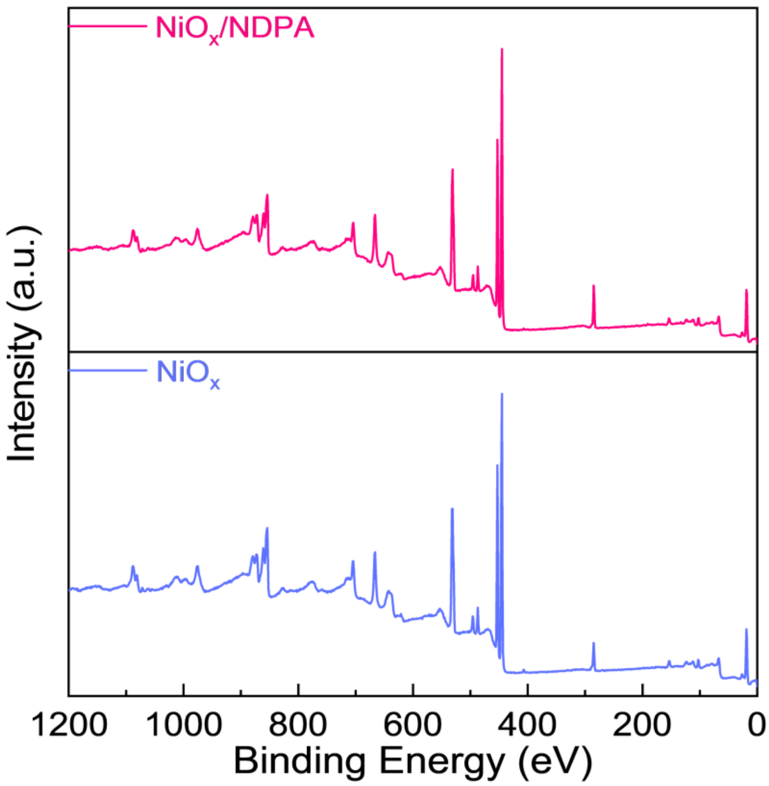

Figure S6. Survey XPS spectra on NiO_x and NiO_x/NDPA substrates.

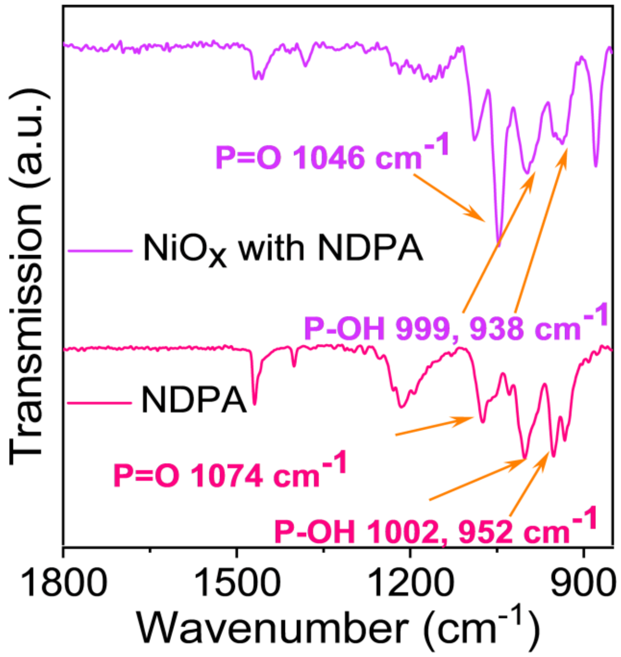

Figure S7. FTIR spectra of the pure NDPA solution and the mixing between NDPA and NiO_x.

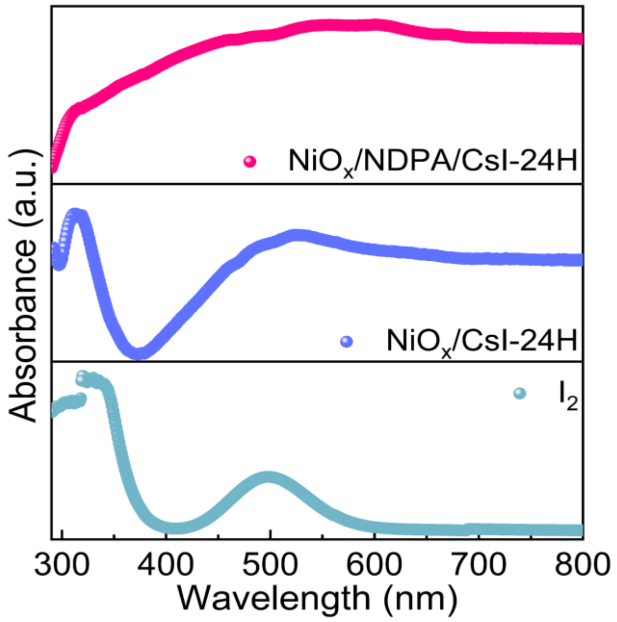

Figure S8. Solution UV-Vis spectra of the I_2, NiO_x/CsI and NiO_x/NDPA/CsI substrates in toluene after storing for 24 hours.

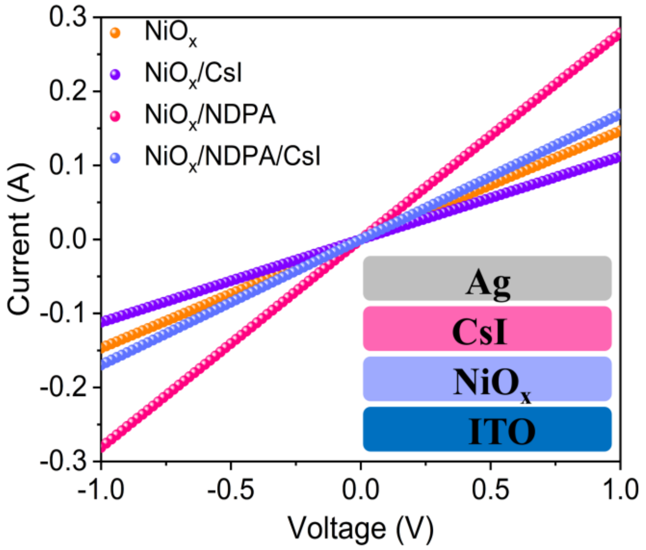

Figure S9. Current-voltage characteristics of NiO_x-based substates.

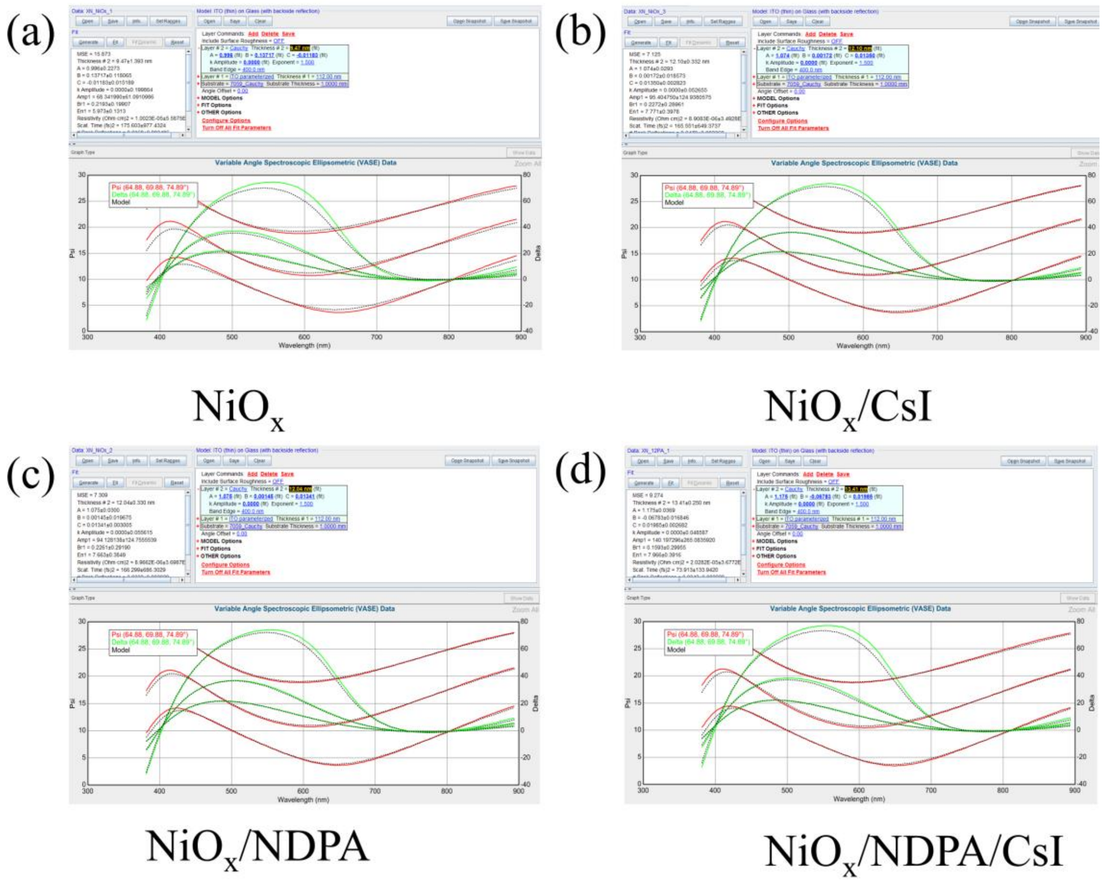

Figure S10. Thickness of NiO_x on different substrates.

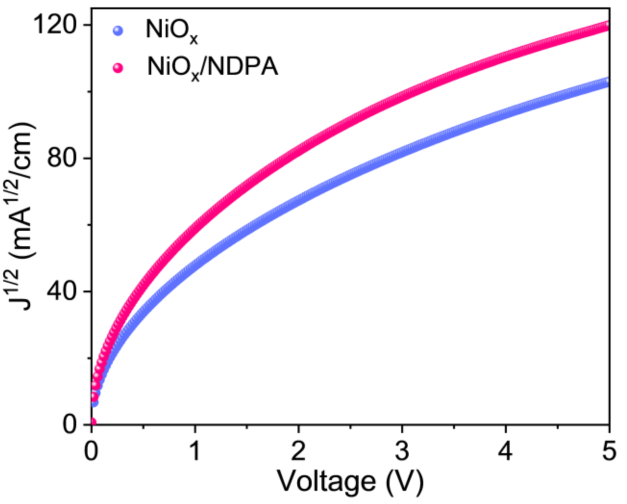

Figure S11. The $\sqrt{J}$-V characteristic of ITO/NiO$_x$ without or with NDPA/Ag hole-extracting devices.

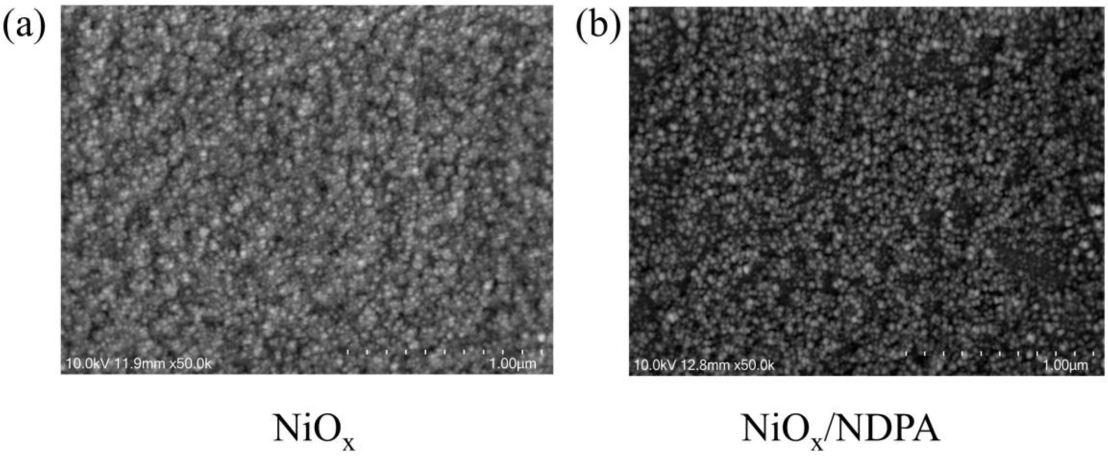

Figure S12. Surface morphology of NiO_x-based substates via SEM measurement.

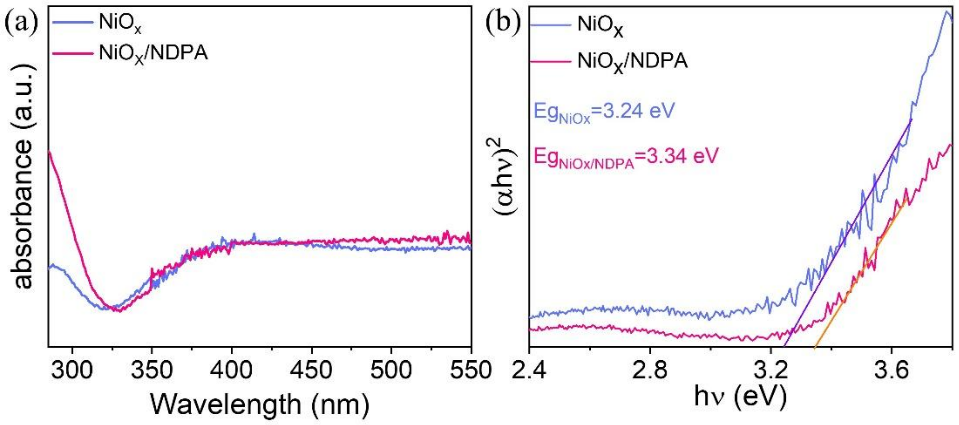

Figure S13. UV-Vis spectra a) and the corresponding Tauc plots b) of the different NiO_x-based substates.

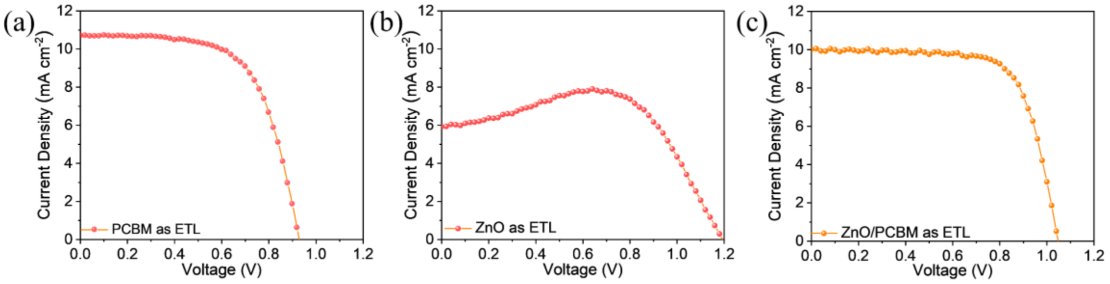

Figure S14. *J*-*V* curves of the $CsPbIBr_2$ PSCs using different ETL.

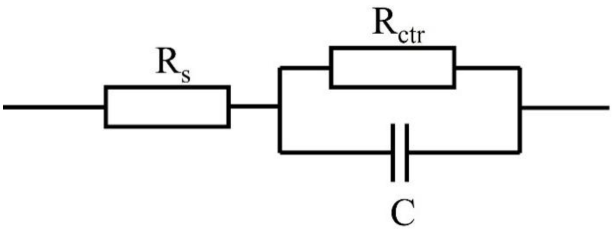

Figure S15. Fitting circuit of the EIS plots.

Table S1 Conductivity of NiO_x-based substrates.

Sample	Thickness (nm)	Electrode area (cm^{-2})	Conductivity ($S\ cm^{-1}$)
NiO_x	9.5	0.06	2.32×10^{-6}
NiO_x/CsI	12.1	0.06	2.22×10^{-6}
NiO_x/NDPA	12.0	0.06	5.58×10^{-6}
NiO_x/NDPA/CsI	13.4	0.06	3.78×10^{-6}

Note: conductivity (σ) was estimated via the following equation[2]: $\sigma = \frac{Id}{AV}$, where *A* is the device area, *d* is the thickness of NiO_x layer which is obtained by ellipsometry as shown in figure S10, *I* is the current, V is the applied voltage.

Table S2 detailed fitting parameters of the pristine and target perovskite films.

Sample	A1	τ_1 (ns)	A2	τ_2 (ns)	τ_{ave} (ns)
Pristine	0.916	0.159	0.226	1.86	1.77
Target	13.4	0.114	0.242	1.40	1.13

Note: TRPL spectrum is calculated using the following equation[3]: $\tau_{ave} = \frac{A_1\tau_1^2+A_2\tau_2^2}{A_1\tau_1+A_2\tau_2}$

Table S3 detailed photovoltaic parameters of different ETL-based $CsPbIBr_2$ PSCs.

Sample	V_{oc} (V)	J_{sc} ($mA\ cm^{-2}$)	FF (%)	PCE (%)
PCBM	0.93	10.7	64.0	6.37
ZnO	1.19	5.29	76.9	5.44
ZnO/PCBM	1.05	10.0	70.7	7.41

Table S4 the statistic photovoltaic performance for NiO_x-based $CsPbIBr_2$ perovskite solar cells.

Device configuration	V_{oc} (V)	J_{sc} ($mA\ cm^{-2}$)	FF (%)	PCE (%)	Reference
FTO/NiO_x/$CsPbIBr_2$/PCBM/BCP/Ag	0.79	4.24	56.40	1.89	[4]
FTO/NiO_x/$CsPbIBr_2$/MoO_x/Au	0.85	10.56	62.00	5.52	[5]
FTO/ NiO_x/$CsPbIBr_2$/ZnO/Al	1.01	8.65	63.60	5.57	[6]
ITO/NiO_x/$CsPbIBr_2$/CeO_x/Ag	1.01	8.76	63.35	5.60	[7]
FTO/Cs-NiO_x/$CsPbIBr_2$/PCBM/BCP/Ag	1.19	11.49	69.00	9.49	[8]
ITO/ NiO_x/$CsPbIBr_2$/ZnO/PCBM/Ag	1.12	11.30	73.43	9.28	This Work

Table S5 fitting parameters of the EIS plots.

Sample	pristine	target
R_s (Ω)	45.2	32.8
R_{ctr} (Ω)	2.2×10^4	2.8×10^4
C (F)	6.05×10^{-8}	5.38×10^{-8}

References

[1] S. Gyawali, L. K. M. O. Goni, M. S. Chowdhury, A. Laref, S. Bajgai, S. Chantrapromma, K. Techato, *Mater. Res. Express* **2022**, *9*, 055004.

[2] X. Chen, C.-H. Chen, Z. H. Su, J. Chen, K.-L. Wang, Y. Xia, N. Nizamani, L. Huang, R.-J. Jin, Y.-H. Li, X. Yu Gao, Z.-K. Wang, *Adv. Funct. Mater.* **2025**, *35*, 2415004.

[3] R. Zhao, L. Xie, R. Zhuang, T. Wu, R. Zhao, L. Wang, L. Sun, Y. Hua, *ACS Energy Lett.* **2021**, *6*, 4209.

[4] W. Chai, W. Zhu, J. Ma, S. Huangfu, Z. Zhang, D. Chen, J. Zhang, C. Zhang, Y. Hao, *Appl. Surf. Sci.* **2022**, *595*, 153544.

[5] C. Liu, W. Li, J. Chen, J. Fan, Y. Mai, R. E. I. Schropp, *Nano Energy* **2017**, *41*, 75.

[6] J. Lin, M. Lai, L. Dou, C. S. Kley, H. Chen, F. Peng, J. Sun, D. Lu, S. A. Hawks, C. Xie, F. Cui, A. P. Alivisatos, D. T. Limmer, P. Yang, *Nature Mater.* **2018**, *17*, 261.

[7] J. Yang, Q. Zhang, J. Xu, H. Liu, R. Qin, H. Zhai, S. Chen, M. Yuan, *Nanomaterials* **2019**, *9*, 1666.

[8] S. Yang, L. Wang, L. Gao, J. Cao, Q. Han, F. Yu, Y. Kamata, C. Zhang, M. Fan, G. Wei, T. Ma, *ACS Appl. Mater. Interfaces* **2020**, *12*, 13931.
