# Supplementary material for: Suppressing NiOx/CsPbIBr2 Interfacial Redox Reactions and Band Energy Misalignment in Perovskite Solar Cells
Source: Small Methods. 2026 Jan 29;10(4):e01684. doi: 10.1002/smtd.202501684 (PMC12929923; doi:10.1002/smtd.202501684)
Supplement: Supplementary file 2 — Supporting File: smtd70514‐sup‐0002‐DataFile.zip. [file SMTD-10-e01684-s001.zip › Solar_Cell_Data_Reporting_Checklist-1663587698650.pdf]

WILEY-VCH

Solar Cell Data Reporting Checklist

At Wiley-VCH, we wish to improve the reproducibility and data quality of our published content according to the FAIR principles. Please fill out this form as part of the submission process for all original research articles reporting efficiency and/or stability-related investigations of photovoltaic devices. For further information please see Wiley-VCH's data sharing policy

**Materials and Performance Parameters of the Champion Cell**	
Photoactive material(s) used	CsPbIBr2 photoacitve material
Please provide the values for power conversion efficiency (PCE [%]), open circuit voltage (V_{OC} [mV]), short circuit current density (J_{SC} [mA/cm^2]), and Fill Factor (FF[%]).	PCE:9.28%,Voc:1120 mV, Jsc:11.3 mA cm-2, FF:73.43%
Is stabilized performance reported (please see *ref.1* for details)?	Stabilized Current at Fixed Voltage (SCFV)
Are current density-voltage (J-V) plots provided in both forward and backward directions (only applicable for perovskite solar cells)?	Yes ◉ No ○
**J-V Measurement of the Champion Cell**	
Please state whether your devices were tested under standard test conditions (T_{cell}: 25°C; J-V incident light intensity: 100 mW/cm^2).	Yes ◉ No ○
Please state the reference spectrum used.	AM1.5G
Was a spectrally adjustable solar simulator used (only applicable for multijunction cells)?	Yes ○ No ◉
Mask aperture area [cm^2]	without
Total device active area [cm^2]	0.06
J-V measurement data file* Inclusion of the J-V plot in the manuscript is mandatory; please indicate whether you have also uploaded* the measurement data file (encouraged)	Yes ◉ No ○
**Bandgap Energy Measurement of the Champion Cell**	
Bandgap Energy E_g [eV] (taken from the EQE spectrum, at the inflection point of the absorption threshold) (please see *ref.2* for details).	2.1

Integrated J_{SC} [mA/cm^2] (taken from the EQE spectrum under 1 sun AM1.5G illumination)	10.1
EQE data file*<br>Inclusion of the EQE spectrum in the manuscript is mandatory; please indicate whether you have also uploaded* the data file (encouraged)	Yes ◉<br>No ○
**Semitransparent PV** (only applicable for transparent/semitransparent devices)	
Average visible transmittance, AVT [%]<br>(please see 'Data S1' in *ref.3* for the calculation)	
Transmittance measurement data file*<br>Inclusion of the transmittance spectrum in the manuscript is mandatory for transparent/ semitransparent devices; please indicate whether you have also uploaded* the data file (encouraged)	Yes ○<br>No ○
Have you confirmed EQE[%]+Transmittance [%]<1?	Yes ○<br>No ○
**Long-Term Stability Test**	
Please provide the initial values for PCE [%], V_{OC} [mV], J_{SC} [mA/cm^2], and FF[%]	PCE: 8.86%, Voc:1129 mV,Jsc: 10.44 mA cm-2, FF: 75.18%
Please state incident light spectrum [e.g., AM1.5G, white LED, etc.], incident illumination intensity (e.g., 1 sun), electrical bias condition (open circuit/MPP/short circuit/constant load), UV filter, test temperature, cell encapsulation, atmosphere composition (air/N_2/Ar), relative humidity [%], etc.<br>(please see 'Table 3' in *ref.4* for more details).	AM 1.5 G

* to be submitted as a separate file when uploading your production data:

J-V measurement data file: two columns csv format: Voltage [V], Current density [mA/cm^2]

EQE data file: two columns csv format: wavelength [nm], EQE [%]

Transmittance measurement data file: two columns csv format: wavelength [nm], T [%]

1. Dunbar, R. B. *et al.* How reliable are efficiency measurements of perovskite solar cells? The first inter-comparison, between two accredited and eight non-accredited laboratories. *J. Mater. Chem. A*, **2017**, 5, 22542.
2. Almora, O. *et al.* Quantifying the Absorption Onset in the Quantum Efficiency of Emerging Photovoltaic Devices. *Adv. Energy Mater.* **2021,** 11, 2100022.
3. Yang, C. *et al.* How to accurately report transparent solar cells. *Joule*, **2019**, 3, 8, 1803-1809.
4. Khenkin, M.V., Katz, E. A., Abate, A. *et al.* Consensus statement for stability assessment and reporting for perovskite photovoltaics based on ISOS procedures. *Nat. Energy*, **2020**, 5, 35– 49.

This Solar Cell Data Reporting Checklist is made available under the Creative Commons Attribution (CC-BY) License, which permits use, distribution, and reproduction in any medium, provided the original checklist is properly cited. Please consult the related article for the license terms applicable to the article.
